# Hyperoxia promotes polarization of the immune response in ovalbumin-induced airway inflammation, leading to a TH_17_ cell phenotype

**DOI:** 10.1002/iid3.71

**Published:** 2015-06-18

**Authors:** Akinori C Nagato, Frank S Bezerra, André Talvani, Beatriz J Aarestrup, Fernando M Aarestrup

**Affiliations:** 1 Laboratory of Immunopathology and Experimental Pathology, Center for Reproductive Biology—CRB, Federal University of Juiz de ForaJuiz de Fora, Minas Gerais, Brazil; 2 Laboratory of Metabolic Biochemistry (LBM); 3 Laboratory of Immunobiology of Inflammation, Department of Biological Sciences (DECBI), Center of Research in Biological Sciences (NUPEB), Federal University of Ouro Preto (UFOP)Ouro Preto, Minas Gerais, Brazil

**Keywords:** Airway inflammation, hyperoxia, immune response, mice

## Abstract

Previous studies have demonstrated that hyperoxia-induced stress and oxidative damage to the lungs of mice lead to an increase in IL-6, TNF-α, and TGF-β expression. Together, IL-6 and TGF-β have been known to direct T cell differentiation toward the TH_17_ phenotype. In the current study, we tested the hypothesis that hyperoxia promotes the polarization of T cells to the TH_17_ cell phenotype in response to ovalbumin-induced acute airway inflammation. Airway inflammation was induced in female BALB/c mice by intraperitoneal sensitization and intranasal introduction of ovalbumin, followed by challenge methacholine. After the methacholine challenge, animals were exposed to hyperoxic conditions in an inhalation chamber for 24 h. The controls were subjected to normoxia or aluminum hydroxide dissolved in phosphate buffered saline. After 24 h of hyperoxia, the number of macrophages and lymphocytes decreased in animals with ovalbumin-induced airway inflammation, whereas the number of neutrophils increased after ovalbumin-induced airway inflammation. The results showed that expression of Nrf2, iNOS, T-bet and IL-17 increased after 24 of hyperoxia in both alveolar macrophages and in lung epithelial cells, compared with both animals that remained in room air, and animals with ovalbumin-induced airway inflammation. Hyperoxia alone without the induction of airway inflammation lead to increased levels of TNF-α and CCL5, whereas hyperoxia after inflammation lead to decreased CCL2 levels. Histological evidence of extravasation of inflammatory cells into the perivascular and peribronchial regions of the lungs was observed after pulmonary inflammation and hyperoxia. Hyperoxia promotes polarization of the immune response toward the TH_17_ phenotype, resulting in tissue damage associated with oxidative stress, and the migration of neutrophils to the lung and airways. Elucidating the effect of hyperoxia on ovalbumin-induced acute airway inflammation is relevant to preventing or treating asthmatic patients that require oxygen supplementation to reverse the hypoxemia.

## Introduction

Asthma is a chronic inflammatory airway disease characterized by shortness of breath, bronchial hyperreactivity, obstruction, and airway remodeling that is induced by cells of the innate and adaptive immune system 1–6. Although initially associated with a TH_2_ response, more severe forms of asthma have been associated with a predominantly neutrophilic response 7, with no contribution from TH_2_ cytokines 8, but instead with a mix of cytokines with TH_1_ and TH_17_ 9. The airway obstructions more severe with worse reversibility. Clinical interventions require oxygen supplementation in supra-physiological concentrations (hyperoxia) to reverse the hypoxemia.

Paradoxically, hyperoxia alone induces pulmonary inflammation, characterized by an influx of neutrophils and macrophages into airways 10, increased expression of cytokines IL-6, TGF-β, and IFNγ 11–14, pulmonary edema, epithelial and endothelial cells death 15, apoptosis, oxidative stress, damage to extracellular matrix proteins 16,17, and DNA 18 and lipid peroxidation 19. The hyperoxia-induced oxidative stress is characterized by a redox imbalance with increased levels of reactive oxygen species (ROS), such as superoxide anion (O_2_^−^) and hydroxyl radical (OH^Ÿ^), and reactive nitrogen species (NOS) nitrite and peroxide (ONOO^−^) 20–25.

Hyperoxia lead to a redox imbalance in inflammatory cells controlled by an antioxidant system 26,27. Activation of nuclear factor (erythroid-derived 2)-like 2 (Nrf2) results in increased expression of the genes of several antioxidant enzymes, including catalase (CAT), superoxide dismutase (SOD), glutathione peroxidase (GPx), reduced glutathione (GSH), oxidized glutathione (GSSG), and myeloperoxidase (MPO) 28,29. Increased levels of ROS and NOS and reduced antioxidant enzyme activities lead to oxidative damage of the cell 30. Oxidative damage can be experimentally detected by monitoring the products of lipid peroxidation, such as malondialdehyde (MDA) 31.

Oxidative stress has also been investigated as a condition favoring perpetuation of inflammation, acute lung injury 32,33, and polarization of lymphocytes 34–36. The mechanisms that trigger activation, proliferation, and differentiation of T cells rely on both coupling with the TCR 37 and activation of specific transcription factors. Studies show that activation of IL-6R, TGF-βR, IL-21R, IL-23R, STAT3, and IFR4 induces differentiation of naive TH cells into TH_17_ cells by activation of RORγt transcription factor 38–40. The TH_17_ cells produce IL-17 and IL-22, recruiting monomorphonuclear and polymorphonuclear cells 41,42. In the pathophysiology of asthma, neutrophil recruitment has been understood to be an aggravating factor in inflammation 43–46. TH_1_-cell polarization depends upon the activation of Toll-like receptor 9 (TLR9), IFNγ, IL-12, STAT4, and Tbet, which leads to induction of the TH_1_ cytokine pattern (INFγ and TNF-α) 47–51. This pathway generates CD8^+^ cytotoxic T cell (T_C_1) and CD4^+^ TH cell responses, and activates mononuclear phagocytes for defense against pathogens. Differentiation into TH_2_ cells is induced by activation of the transcription factor GATA3, triggered by the activation of STAT6, STAT5, IL-2R, and IL-4R 52–57. TH_2_ cell activation results in IL-4, IL-5, and IL-13 production, activating mast cells, basophils, and eosinophils.

Changes in T cells populations are also marked by the cytokines expressed by lymphocytes and dendritic cells during inflammation. It has been shown that the reduced expression of CCL2 (MCP-1), for example, results in the smallest TH_2_ response 58. On the other hand, the overexpression of CCL5, and the activations of GATA3 and STAT6 induce a robust TH_2_ phenotype 59.

The ovalbumin-induced airway inflammation in BALB/c mice has been widely used as an experimental model of asthma been mediated by TH_1_ 60,61, TH_2_, and TH_17_ 62 cells and induces eosinophil and neutrophil recruitment, goblet cell hyperplasia, increased mucus production, and airway obstruction 63–65.

Previous studies have demonstrated that hyperoxia-induced stress and oxidative damage to the lungs of mice lead to an increase in IL-6, TNF-α, and TGF-β expression 11,12 all together associated to the T cell differentiation toward the TH_17_ phenotype 66,67. In the current study, we tested the hypothesis that hyperoxia promotes the polarization of T cells to the TH_17_ cell phenotype in response to ovalbumin-induced acute airway inflammation.

## Methods

### Animals

Female BALB/c mice (6–8 weeks, 20–25 g) were purchased from the Central Biotherium of the Federal University of Ouro Preto (Ouro Preto-MG/Brazil) in a controlled-environment with cycled lighting (12 h light/12 h dark, lights on at 6:00 PM), with controlled temperature (21−22°C ± 2°C) and relative humidity (50–55%). The animals received food and water ad libitum. During hyperoxia studies, water bottles (60 mL) and triturated feed troughs were positioned inside the chamber. The study was conducted in accordance with Brazilian Federal Guidelines for Laboratory Animal Use and Care (Brazilian Law 11.794 from August 12, 2008). The experimental design was approved by Ethics Committee for Animal Research of UFOP (N^0^. 107/2012).

### Induction of acute airway inflammation and hyperoxia protocols

**Figure 1:**
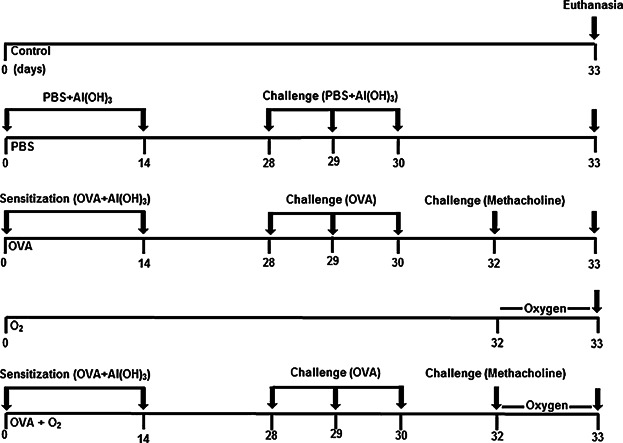
Experimental timeline of the ovalbumin (OVA)-induced acute airway inflammation and hyperoxia six-week-old female BALB/c mice model. Control (air room)—Control mouse exposed to normoxia. PBS + Al(OH) 3—Mice were challenged with aluminium hydroxide (Al(OH) 3) in phosphate buffered saline (PBS) by intraperitoneal injections (on days 0 and 14), following challenged by intranasal PBS + Al(OH) 3 administration (days 28, 29, and 30). OVA—Mice were sensitized with ovalbumin (OVA) by intraperitoneal injections (on days 0 and 14), following challenged by intranasal OVA administration (days 28, 29, and 30) and nebulized methacholine (On 32 day). O_2_—The animals were exposed to 100% oxygen in chamber for 24 h. For more details, see the Materials and Methods section. After the euthanasia, bronchoalveolar lavage was collected and lungs were removed for analysis.

Sensitization was induced in OVA (*n* = 06) and OVA + O_2_ (*n* = 06) groups by intraperitoneal injection of 20 mg ovalbumin in 2 mg aluminum hydroxide (Al(OH)_3_) dissolved in 200 µL phosphate buffered saline, pH 7.4 (PBS). Airway inflammation was induced by intranasal introduction of 50 μL of 1% ovalbumin, followed by challenge with 2.5% nebulized methacholine for 20 min. After the methacholine challenge, the animals were exposed to hyperoxic conditions in an inhalation chamber (length = 30 cm, width = 20 cm, and height = 15 cm) as previously described 11,17,19. Medical-grade oxygen was purchased from White Martins (Praxair, Inc., São Paulo, Brazil). The O_2_ (*n* = 06) and OVA + O_2_ (*n* = 06) groups received continuous oxygen at 10 L/min for 24 h (Fig. 1).

### Bronchoalveolar lavage (BAL)

Analgesic and sedative (100 mg/kg ketamine and 15 mg/kg xylazine). After hyperoxic treatment, they were immediately euthanized by cervical dislocation. Lungs and airways were washed three times with 500 µL PBS (final wash volume was 1.2–1.5 mL). The left lung was collected, homogenized in 1 mL cold PBS, centrifuged at 345,881*g* (7500 rpm for 10 min), and supernatant were collected and stored at −86°C for biochemical analysis.

### Cell analysis of BAL

The total countage of cells in the bronchoalveolar lavage was performed using a Neubauer chamber and the differential countage using cytospin technique (Panotic stained with Fast). The identification of inflammatory cells and differential counts were performed on slides by light microscopy (magnitude 1000×).

### Biochemical analysis

All chemicals were purchased from Sigma–Aldrich Chemical Co., (Sigma-Aldrich Inc., St. Louis, MO, USA). All measurements described below were performed on lung homogenates using a spectrophotometer (Beckman model DU 640; Fullerton, CA) or a microplate reader (Bio-Rad model 550, Hercules, CA). Catalase (CAT) activity was calculated from the rate of decrease in the concentration of hydrogen peroxide, which was determined from the absorbance at 240 nm 68. The level of malondialdehyde (MDA) was measured during an acid-heating reaction with thiobarbituric acid, and was determined from the absorbance at 532 nm 69. The total protein contents in the BALs and lung tissue homogenates were determined according to the Bradford method 70.

### Immunohistochemical analysis

Slices with 4 µm thickness were arranged on silanized slides (3-aminopropyltriethoxysilane; Sigma-Aldrich Inc., St. Louis, MO, USA), deparaffinized in a 60°C chamber, sequentially hydrated in passages through xylol, absolute alcohol, 70% alcohol, and distilled water. The avidin-biotin peroxidase-anti-peroxidase complex method was used for sample staining. Tissue samples were immersed in 1 mM citrate buffer, PH 6.0, blocked with 3% hydrogen peroxide, and incubated with primary antibodies (polyclonal rabbit anti-iNOS, anti-Nrf2, anti-T-bet, and anti-IL17, diluted 1:100—Santa Cruz, CA) for 1 h. Next, samples were incubated with biotinylated secondary antibodies for 30 min and the avidin-biotin complex for an additional 30 min. Samples were stained by addition of the diaminobenzidine chromogen (DAB) substrate for ∼1 min. The negative control were carried out by omitting incubation with primary antibodies. Tissue sections were examined by light microscopy (400×), and 10 photomicrographs were collected per section (Zeiss Axiostar, Zeiss, Germany).

### Histopathological, histomorphometric, and stereologic analysis

Five-micron-thick right lung tissue sections were stained with hematoxylin and eosin (H&E) and examined by light microscopy at 100×, 400×, and 1000× magnification. Ten photomicrographs/lung sections were analyzed from representative fields using an image capture system (AxioCam CHF 5—Carl Zeiss Microscopy GmbH, 2011; Zeiss, Berlin, Germany). The pulmonary parenchyma area density (Vv_par_) in each field was calculated by subtracting the area occupied by air space (A_air_) from the microscopic field total area (A_image_), then: (Vv_par_= A_image_−A_air_). The mean occupancy by lung parenchyma in µm^2^ was corrected for each group by the magnitude in square millimeters. The images were stored and submitted to identification of inflammatory cells influx and presence macrophages, neutrophils, lymphocyte, and eosinophils in lung parenchyma or alveolar spaces.

In each pulmonary section, the positive immunostaining density (for iNOS, Nrf2, T-bet, and IL-17) was measured by brown tones density of immunostained chromogen (µm^2^). Immunostaining density was corrected by lung parenchyma area density.

### ELISA measurements of chemokines: TNF-α, CCL2, and CCL5

The content of TNF-α, CCL2, and CCL5 was analyzed from the lung homogenate supernatant 71. Supernatant was combined with the inflammatory fluid, and measured by ELISA using kits from R&D systems. Briefly, flat-bottom 96-well microtiter plates (Nunc) were coated with 100 μL/well of the appropriate monoclonal antibodies for 18 h at 4°C, and washed with wash buffer (1 × PBS, 0.05% Tween-20). Nonspecific binding sites were blocked with 200 μL/well of blocking buffer (1% BSA in PBS). Plates were rinsed with wash buffer and appropriately diluted samples were added (100 μL/well), followed by incubation for 18 h at 4°C. Plates were then washed and 100 μL/well of the appropriate biotinylated detection antibodies diluted in blocking buffer containing 0.05% Tween-20 were added for 1 h at room temperature. Plates were then washed, streptavidin-horseradish peroxidase was added (100 μL/well), and plates were incubated for 30 min at room temperature. Plates were then washed, 100 μL/well of the chromogen substrate OPD (o-phenylendiamine, Sigma-Aldrich Inc., St. Louis, MO, USA) in 30 mM citrate buffer (pH 5.0) containing 0.02% v/v H_2_O_2_ was added, and the plates were incubated in the dark for 30 min at room temperature. The reaction was terminated with 50 μL/well of 1 M H_2_SO_4_. Plates were read at 492 nm in a spectrophotometer (E max—Molecular Devices). All samples were assayed in duplicate. The threshold of sensitivity for each chemokine is 15.625 pg/mL.

### Statistical analysis

The data are presented as the mean ± standard error of the mean (SEM). For continuous data, we used a one-way ANOVA followed by the Student–Newman–Keuls post hoc test (for CAT, MDA, protein, BAL, and morphometry results). For non-continuous data, we used the Kruskal–Wallis test followed by the Dunn's post hoc test (stereology results). In all instances, the significance level was set at 5% (*P* < 0.05). Analyses were performed from GraphPad Prism version 5:00 for Windows (GraphPad Software, San Diego, CA).

## Results

### Cellular profiles of BAL after ovalbumin-induced airway inflammation and hyperoxia

**Figure 2:**
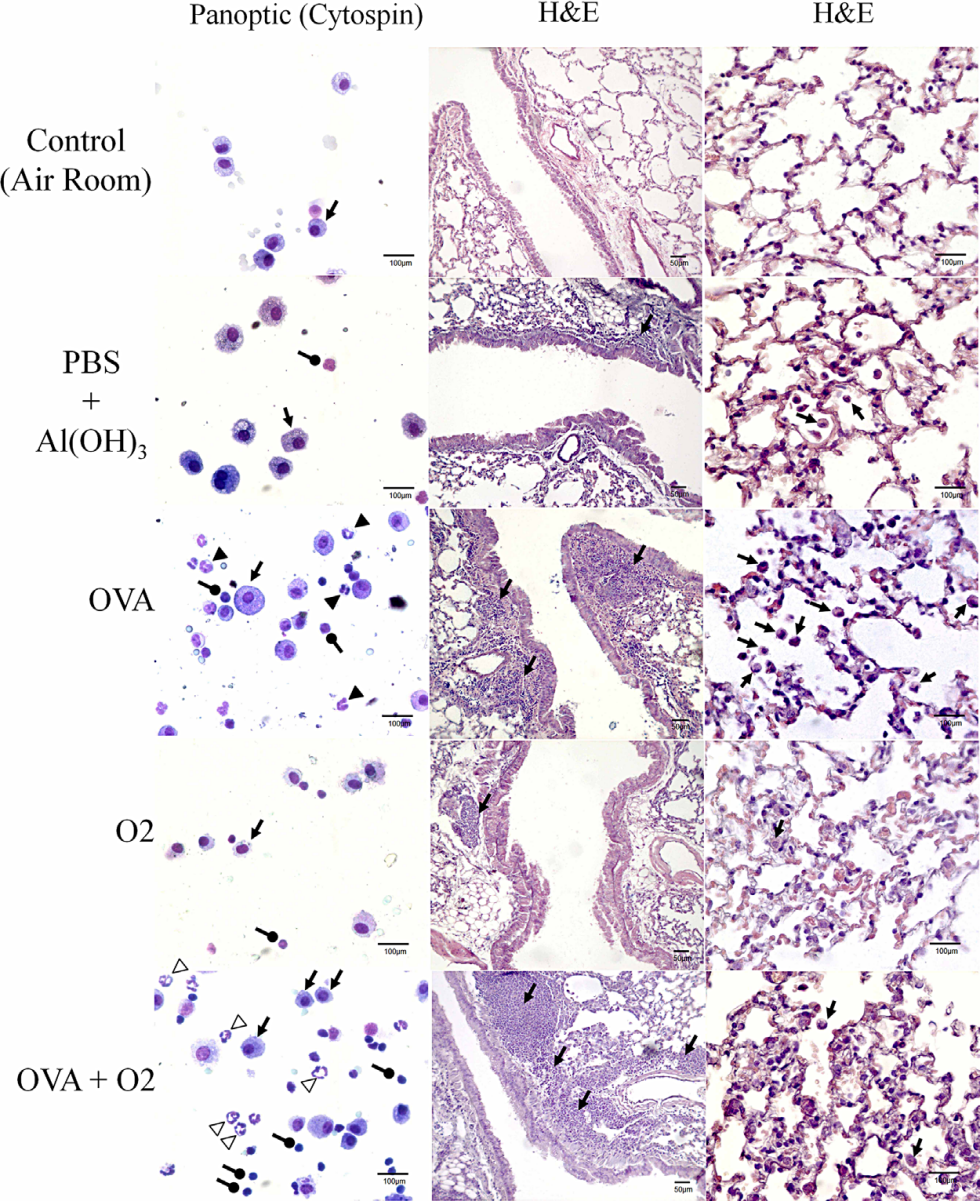
The profile of inflammatory cells in bronchoalveolar lavage (BAL) and lung histopathology (H&E) after ovalbumin (OVA)-induced acute airway inflammation and (O_2_) hyperoxia. Control (air room)—Control mouse exposed to normoxia. PBS + Al(OH) 3—Mice were challenged with aluminium hydroxide (Al(OH) 3) in phosphate buffered saline (PBS) by intraperitoneal injections (on days 0 and 14), following challenged by intranasal PBS + Al(OH) 3 administration (days 28, 29, and 30). OVA—Mice were sensitized with ovalbumin (OVA) by intraperitoneal injections (on days 0 and 14), following challenged by intranasal OVA administration (days 28, 29, and 30) and nebulized methacholine (On 32 day). O_2_—The animals were exposed to 100% oxygen in chamber for 24 h. For more details, see the Materials and Methods section. (Left Column Panoptic—Cytospin)—Lymphocytes (circular arrows), macrophages (arrows), eosinophils (black arrowhead), and neutrophils (white arrowhead). (scale bar, 100 µm). (H&E)—Haematoxylin and eosin staining. (Middle column H&E)—Lung section show peribronchial and perivascular accumulation of inflammatory cells (arrows) more pronounced in OVA and OVA + O_2_ groups (scale bar, 50 µm). (Right column H&E)—H&E-stained parenchyma lung sections (5 µm thick) show many alveolar macrophage in OVA group and a pronounced volume density of lung parenchyma. (scale bar, 100 µm).

**Figure 3:**
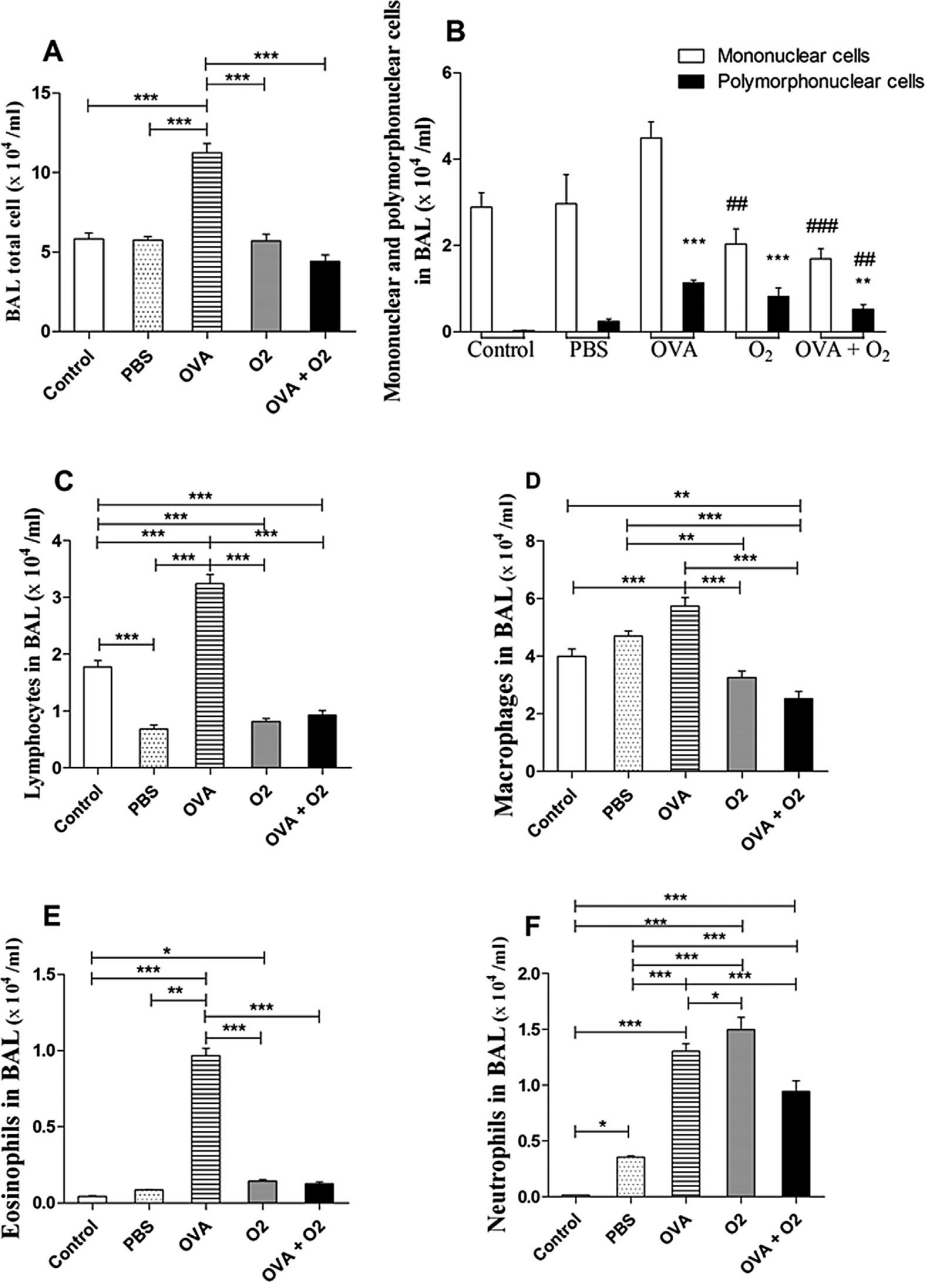
Effects of hyperoxia on number of inflammatory cells. (A) Bronchoalveolar lavage (BAL) total cell recovery. (B) Mononuclear and polymorphonuclear differential cell recovery. (C–F) Differential cell recovery. Cell counts performed in BAL by using neubauer chamber; differential cell counts were performed on cytospin preparations. Control (air room)—Control mouse exposed to normoxia. PBS + Al(OH) 3—Mice were challenged with aluminium hydroxide (Al(OH) 3) in phosphate buffered saline (PBS) by intraperitoneal injections (on days 0 and 14), following challenged by intranasal PBS + Al(OH) 3 administration (days 28, 29, and 30). OVA—Mice were sensitized with ovalbumin (OVA) by intraperitoneal injections (on days 0 and 14), following challenged by intranasal OVA administration (days 28, 29, and 30) and nebulized methacholine (On 32 day). O_2_—The animals were exposed to 100% oxygen in chamber for 24 h. For more details, see the Materials and Methods section. Values are the means ± standard error of the mean. We used a one-way ANOVA followed by the Tukeýs Multiple Comparison post hoc test. In all instances, significance levels were set at 5%. *P < 0.05 compared to the control group; **P < 0.01 compared to the control group; ***P < 0.001 compared to the control group. ^#^*P* < 0.05 compared to the control group; ^##^*P* < 0.01 compared to the control group; ^###^*P* < 0.001 compared to the control group. *n* = 06 per group.

To determine the effect of hyperoxia on inflammatory cells in the airways following ovalbumin-induced inflammation, we performed a differential count of mononuclear and polymorphonuclear cells in BAL (Fig. 2; Cytospin). When compared to the control group (ambient air), ovalbumin exposure lead to increased recruitment of eosinophils (*P* < 0.001), lymphocytes (*P* < 0.001), macrophages (*P* < 0.001), and neutrophils (*P* < 0.001) to the airways (Fig. 3). In addition, hyperoxia alone (O_2_) significantly increased the number of neutrophils (Fig. 3f; *P* < 0.001). Compared with the ovalbumin-only group (OVA), 24 h after hyperoxia-induced inflammation after ovalbumin (OVA + O_2_), we observed lower numbers of eosinophils (Fig. 3c; *P* < 0.001), lymphocytes (Fig. 3d; *P* < 0.001), macrophages (Fig. 3e; *P* < 0.001), and neutrophils (Fig. 3f; *P* < 0.001).

### Lung histopathology, morphometry, and stereology of lung tissue following ovalbumin-induced airway inflammation and hyperoxia

Analysis of lung tissues from the hyperoxia-induced inflammation after ovalbumin (OVA + O_2_) mice revealed a robust extravasation of inflammatory cells into the lung tissues, particularly into perivascular and peribronchial regions of the lungs, compared to inflamed lungs by ovalbumin only (OVA; Fig. 2, HE column). The lung parenchyma density increased in the PBS control group (0.02 ± 3.95 mm^2^; *P* < 0.05), OVA (0.02 ± 3.97 mm^2^; *P* < 0.01), and O_2_ (12.02 ± 3.97 mm^2^; *P* < 0.01), when compared to the untreated control group (3.85 ± 0.02 mm^2^). However, the differences were even higher when comparing the OVA + O_2_ mice to controls (4.01 ± 0.02 mm^2^; *P* < 0.001). An inverse relationship was observed when measuring the air space density.

### TNF-α, CCL2, and CCL5 content in supernatant of lung homogenate after ovalbumin-induced airway inflammation and hyperoxia

**Figure 4:**
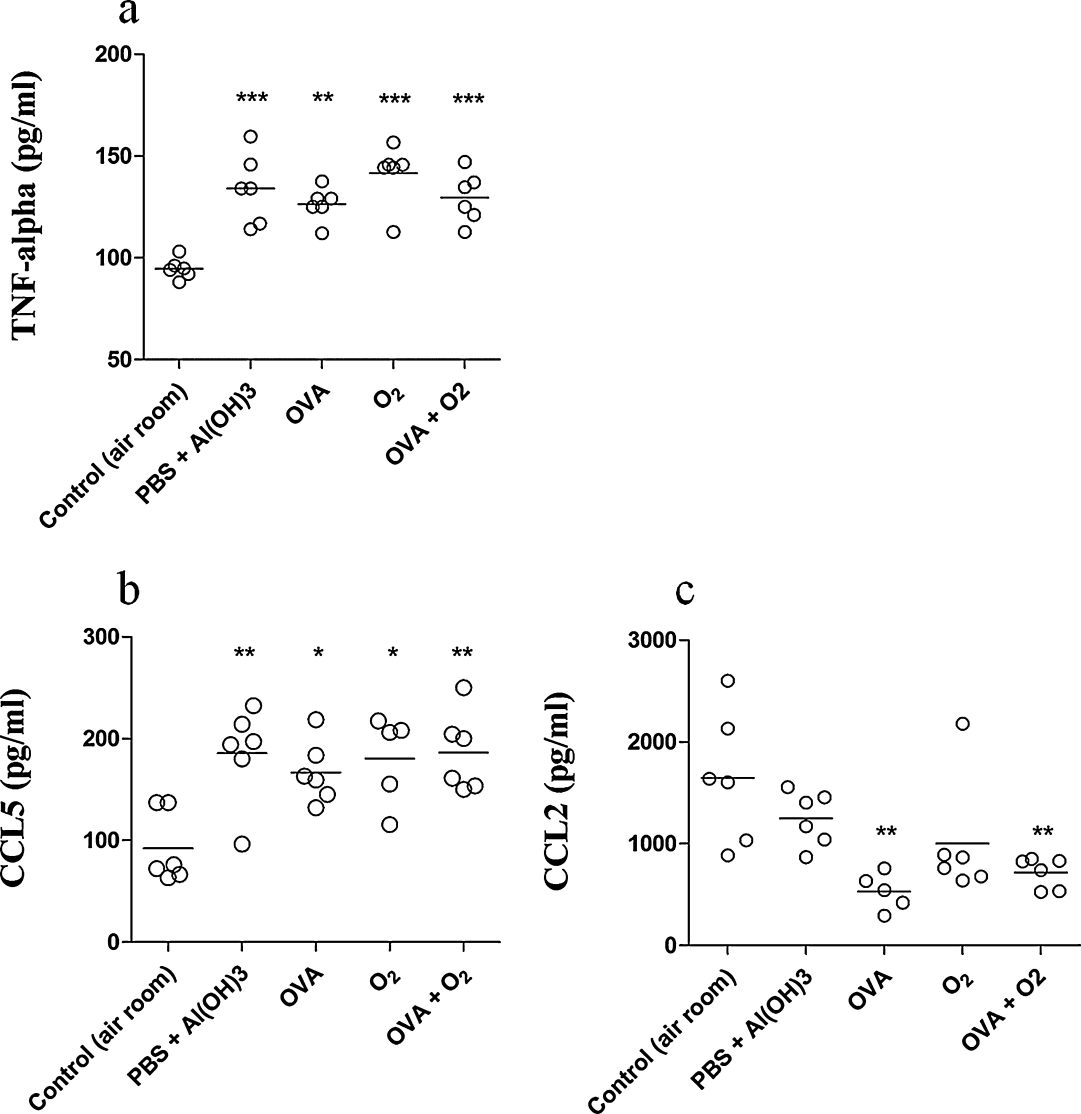
TNF-α, CCL2, and CCL5 content in supernatant of lung homogenate after ovalbumin-induced airway inflammation and hyperoxia. Control (air room)—Control mouse exposed to normoxia. PBS + Al(OH) 3—Mice were challenged with aluminium hydroxide (Al(OH) 3) in phosphate buffered saline (PBS) by intraperitoneal injections (on days 0 and 14), following challenged by intranasal PBS + Al(OH) 3 administration (days 28, 29, and 30). OVA—Mice were sensitized with ovalbumin (OVA) by intraperitoneal injections (on days 0 and 14), following challenged by intranasal OVA administration (days 28, 29, and 30) and nebulized methacholine (On 32 day). O_2_—The animals were exposed to 100% oxygen in chamber for 24 h. For more details, see the Materials and Methods section. We used a one-way ANOVA followed by the Bonferronís Multiple Comparison post hoc test. In all instances, significance levels were set at 5%. *P < 0.05 compared to the control group; **P < 0.01 compared to the control group; ***P < 0.001 compared to the control group.

The lung homogenate supernatant was analyzed by ELISA to verify that hyperoxia caused changes in the levels of TNF-α, CCL2, and CCL5. Hyperoxia alone without the induction of airway inflammation (Group O_2_) lead to increased levels of TNF-α (*P* < 0.001) and CCL5 (*P* < 0.01) in homogenized lung samples, compared to the control group (Fig. 4a, b). The OVA and OVA + O_2_ groups both revealed a significant reduction of CCL2 levels, when compared to the control group (Fig. 4c). Thus, hyperoxia did not potentiate the increase or decrease of TNF-α, CCL2, or CCL5 levels after airway inflammation induced by ovalbumin.

### Hyperoxia-induced oxidative damage after ovalbumin-induced airway inflammation

To determine the oxidative damage induced by hyperoxia following ovalbumin-induced airway inflammation, we determined the malondialdehyde content by spectrophotometry. Malondialdehyde levels were significantly higher (*P* < 0.05) when hyperoxic conditions were introduced to already inflamed airways (Group OVA + O_2_; Table1). Under these conditions, the CAT did not influence hyperoxia on balance control redox.

**Table 1:** Biochemical analysis of oxidative and damage stress in lung homogenate from BALB/c mice exposed to 100% oxygen after ovalbumin-induced allergic airway inflammation

Groups	Control	Vehicle	OVA	O_2_	OVA+O_2_
CAT activity (U/mg ptn)	0.60 ± 0.10	0.53 ± 0.08	0.42 ± 0.03	0.61 ± 0.06	0.79 ± 0.11
MDA (mU/mg ptn)	0.53 ± 0.06	0.25 ± 0.02*	0.63 ± 0.06^#^	0.47 ± 0.05	0.85 ± 0.04^*^^*^^‡^
Protein (μg/μL)^1^	0.12 ± 0.00	0.16 ± 0.01*	0.17 ± 0.00^*^^*^	0.16 ± 0.01*	0.16 ± 0.00*

CAT, catalase; MDA, malondialdehyde. Control group (Control) remained to air room; Vehicle group received aluminum hydroxide in phosphate buffered saline (PBS); OVA group (OVA) received ovalbumin and aluminum hydroxide in PBS; O_2_ group (O_2_) was exposed to 100% oxygen in a chamber for 24 h, and received aluminum hydroxide in PBS; OVA+O_2_ group (OVA+O_2_) received ovalbumin and aluminum hydroxide in PBS and was exposed to 100% oxygen for 24 h. Values are the means ± standard error of the mean.

^*^P < 0.05 compared to the control group. ^*^^*^P < 0.01 compared to the control group. ^‡^*P* < 0.05 compared to the O_2_ group. ^#^*P* < 0.05 compared to the vehicle group. ^##^*P* < 0.01 compared to the vehicle group. For statistics analysis was used the Kruskal–Wallis test followed by the Dunn's post hoc test. In all instances, the significance level was set at 5%. *n* = 06/group.

### Expression of transcription factor Nrf2, inducible nitric oxide synthase (iNOS), T-bet, and IL-17 under conditions of hyperoxia following ovalbumin-induced airway inflammation

**Figure 5:**
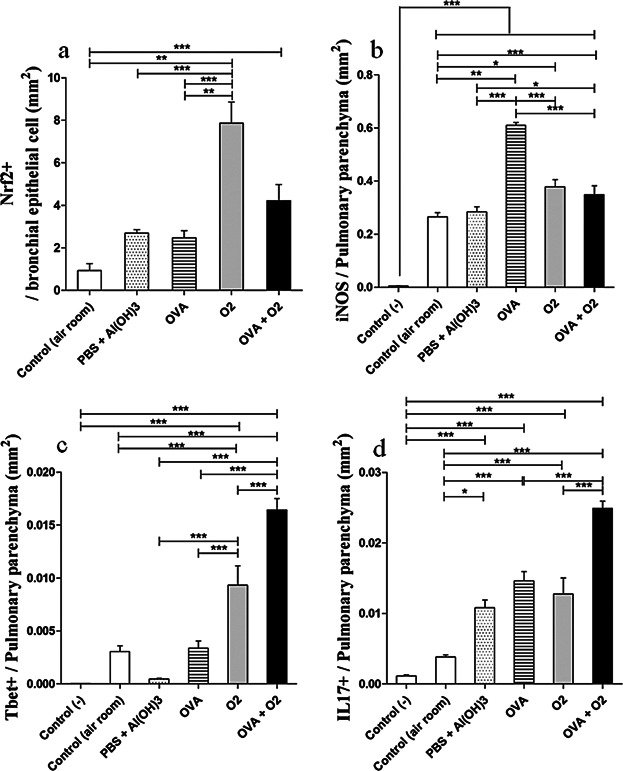
Stereological and Morphometric analysis of bronchial epithelial cells or lung parenchyma cells stained positively (3,3′-Diaminobenzidine [DAB] chromogen) by immunohistochemistry. In ‘a’—Nrf2+/bronchial epithelial cells corresponds to the ratio of brown marked area (Nrf2 positive) per total area of bronchial epithelial cells. “b–d”—iNOS, Tbet+, or IL17+ / pulmonary parenchyma corresponds to the ratio of brown marked area (iNOS, Tbet, or IL17 positive) per total area of pulmonary parenchyma. Area density was performed using a 40× objective lens as described above. Control (−)—As a negative control, the PBS solution was substituted for the primary antibody. Control (air room)—Control mouse exposed to normoxia. PBS + Al(OH) 3—Mice were challenged with aluminium hydroxide (Al(OH) 3) in phosphate buffered saline (PBS) by intraperitoneal injections (on days 0 and 14), following challenged by intranasal PBS + Al(OH) 3 administration (days 28, 29, and 30). OVA—Mice were sensitized with ovalbumin (OVA) by intraperitoneal injections (on days 0 and 14), following challenged by intranasal OVA administration (days 28, 29, and 30) and nebulized methacholine (On 32 day). O_2_—The animals were exposed to 100% oxygen in chamber for 24 h. For more details, see the Materials and Methods section. Values are the means ± standard error of the mean. We used a one-way ANOVA followed by the Bonferronís Multiple Comparison post hoc test. In all instances, significance levels were set at 5%. *P < 0.05 compared to the control group; **P < 0.01 compared to the control group; ***P < 0.001 compared to the control group.

**Figure 6:**
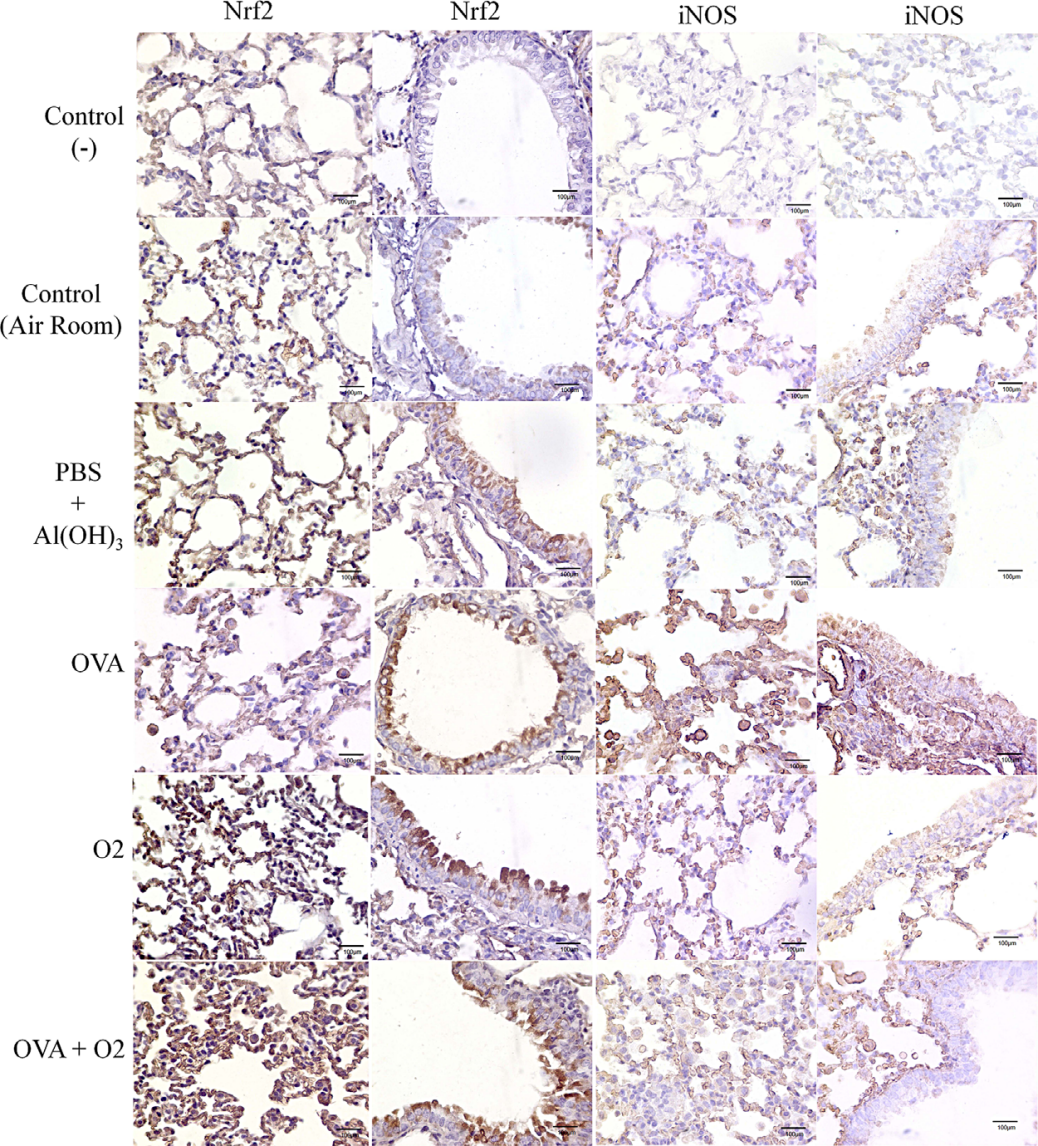
Immunohistochemical analysis was used to localize transcription factor Nrf2 and inducible nitric oxide synthase (iNOS) in lung parenchyma under conditions of hyperoxia following ovalbumin-induced airway inflammation. Control (−)—As a negative control, the PBS solution was substituted for the primary antibody. Control (air room)—Control mouse exposed to normoxia. PBS + Al(OH) 3—Mice were challenged with aluminium hydroxide (Al(OH) 3) in phosphate buffered saline (PBS) by intraperitoneal injections (on days 0 and 14), following challenged by intranasal PBS + Al(OH) 3 administration (days 28, 29, and 30). OVA—Mice were sensitized with ovalbumin (OVA) by intraperitoneal injections (on days 0 and 14), following challenged by intranasal OVA administration (days 28, 29, and 30) and nebulized methacholine (On 32 day). O_2_—The animals were exposed to 100% oxygen in chamber for 24 h. For more details, see the Materials and Methods section. All photomicrograph of negative control shows absence of brown color. A weak (pale) brown color corresponds to a less Nrf2 or iNOS expression. An intense (dark brown) corresponds to a major Nrf2 or iNOS expression.

The transcription factor Nrf2 is activated during oxidative stress, and has been investigated as an important marker of oxidative stress, since it induces the activation of antioxidant enzyme levels when ROS and NOS are high. We investigated the expression of Nrf2 in lung parenchyma by immunohistochemistry. Hyperoxia leads to a significant increase (*P* < 0.01) of Nrf2 expression in airways, alone, or when hyperoxic conditions are introduced after the ovalbumin-induced inflammation (*P* < 0.001), when both are compared with the control group (room air; Figs. 5a, 6).

Hyperoxia alone (O_2_; *P* < 0.05) or after inflammatory condition (OVA + O_2_; *P* < 0.001) both lead to an increased expression of iNOS in lung tissues, compared to the control group (*P* < 0.05; Figs. 5b, 6). In mice, where hyperoxia was maintained for 24 h after inflammation, the ovalbumin-induced expression of iNOS was reduced (O_2_ + OVA group; *P* < 0.001) in the airways, when compared to the OVA group.

There is still uncertainty about the cell profile in ovalbumin-induced inflammation during the acute phase. To address this uncertainty, we investigated whether hyperoxia-induced inflammation after ovalbumin OVA caused modified expression of in T-bet, since this transcription factor induces differentiation of naïve TH lymphocytes to TH1. There was an increased level of T-bet expression in lung tissues related to hyperoxia (O_2_; *P* < 0.001) and hyperoxia after inflammation (O_2_ + OVA; *P* < 0.001) compared to controls (Fig. 5c).

**Figure 7:**
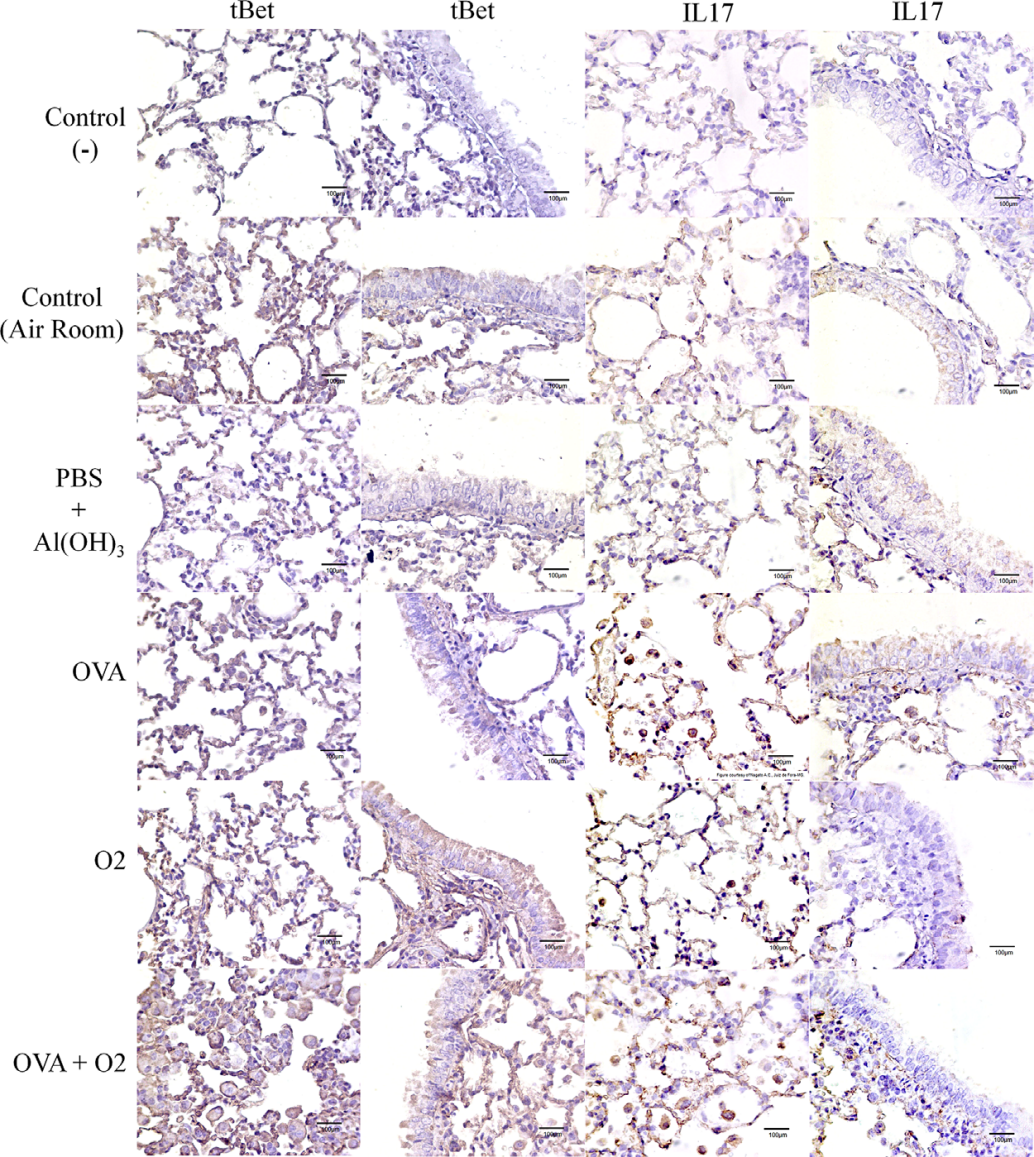
Immunohistochemical analysis was used to localize Transcription factors T-bet, and IL17 in lung parenchyma under conditions of hyperoxia following ovalbumin-induced airway inflammation. Control (−)—As a negative control, the PBS solution was substituted for the primary antibody. Control Air Room—Control mouse exposed to normoxia. PBS + Al(OH) 3–Mice were challenged with aluminium hydroxide (Al(OH) 3) in phosphate buffered saline (PBS) by intraperitoneal injections (on days 0 and 14), following challenged by intranasal PBS + Al(OH) 3 administration (days 28, 29, and 30). OVA—Mice were sensitized with ovalbumin (OVA) by intraperitoneal injections (on days 0 and 14), following challenged by intranasal OVA administration (days 28, 29, and 30) and nebulized methacholine (On 32 day). O_2_—The animals were exposed to 100% oxygen in chamber for 24 h. For more details, see the Materials and Methods section. All photomicrograph of negative control shows absence of brown color. A weak (pale) brown color corresponds to a less T-bet or IL17 expression. An intense (dark brown) corresponds to a major T-bet or IL17 expression.

Besides, it was observed that hyperoxia in inflamed airways significantly increased IL-17 expression (Fig. 5; *P* < 0.001). In addition, an increased expression of IL-17 in both alveolar macrophages and in lung epithelial cells (Fig. 6) was also observed in comparison, compared with both animals that remained in room air and in those with ovalbumin-induced airway inflammation (Fig. 7).

## Discussion

The primary goal of emergency treatment in acute asthma is to reverse the hypoxemia induced by severe airway obstruction. Hypoxemia is treated with administration of oxygen at hyperoxic levels, although it causes toxicity in those vulnerable patients 72. Mice sensitized with ovalbumin are considered a good models to reproduce the hyper-responsive airways observed in human 73.

In this context, hyperoxia supplied for 24 h in mice undergoing OVA-induced acute airway inflammation was able to promote oxidative damage, TH17 cell polarization, and decrease in the number of inflammatory cells (lymphocytes, macrophages, and neutrophils) in the airways. In the pathogenesis of allergic asthma (without hyperoxia), allergens, pollutants, and infectious agents induce GATA-3 transcription factor activation and chemokine CCL2 (MCP-1) expression which promote T cell differentiation toward TH2 and activation of macrophages M2. The TH2 cells secrete cytokines that attract eosinophils (by IL-3, GM-CSF, IL-4, IL-5, and IL-13), which causes damage to the respiratory epithelium by releasing proteases (e.g., eosinophilic proteases) 74–77. On the other hand, when T-bet transcription factor is activated, TH1 cells attract and activate M2 macrophages and neutrophils through soluble mediators such as TNF-α, IL-8, and IL-17A 8,74. Neutrophils are known to cause extensive damage to respiratory epithelium through the release of elastase and metalloproteinases. These enzymes also cause damage to collagen III, essential constituent of the lung extracellular matrix 78–80. Recent studies show that TH17 cells (which secrete IL-17A, IL-17F, IL-22, and IL-26, linfotocin- β, and TNF-α) 81–87 aggravate pulmonary inflammation by the recruitment of neutrophils to the site of inflammation 36. We particularly note factors capable to drive toward the TH1 phenotype in our used experimental model: reduced levels of CCL2, while T-bet, CCL5 (RANTES), and TNF-α levels were elevated. Other study corroborates our findings pointing to the preferential polarization of the immune inflammatory response to CCL5 toward TH1, which also favored the immunological memory 88.

Supported by other studies, we demonstrated that even in the absence of hyperoxia, the number of eosinophils, lymphocytes, macrophages, and neutrophils increased during OVA-induced acute inflammation 5,89–93. However, ovalbumin-induced acute airway inflammation was not able to induce a redox imbalance, as indicated by increased Nrf2 expression in the lungs. By contrast, Nrf2 levels were unchanged, comparable to those of animals that remained in ambient air. Possible, in this context, antioxidant enzymes may have restored redox equilibrium, since the activity of some enzymes (such as CAT) remained unchanged. In our previously study, we also shown that CAT activity was not modified when an important oxidative stress occurred during hyperoxia-induced airway inflammation 11.

Hyperoxia condition causes a redox imbalance in the airways, supported by studies in BALB/c mice and Wistar rats 11,19,94–96. Here, this redox imbalance was reinforced by the increasing of Nrf2 expression in OVA + O_2_ and O_2_ groups. The hyperoxia alone reduces the activity of the enzyme SOD [96 and GSH/GSSG ratio, increased MPO activity and reduced GPx and CAT 11,17,19. It was observed that GPx1 deficient mice have attenuated inflammation by suppression of TH1 and TH17 cells 37.

Oxidative stress has often been investigated in the pathophysiology of asthma in experimental models, where the system of NADPH oxidase activated granulocytes has been touted as the most important source of ROS 98. As a protective measure, the ROS and NOS induce activation of Nrf2 99, and the cells begin to transcribe more antioxidant enzyme genes. In the absence of this activation, there is an increased severity of asthma and inflammation of the airways in mice model 100. Besides, Potteti et al. demonstrated that chronic hyperoxia does not involve the Nrf2 signaling pathways 101. In the present study, the highest levels of Nrf2 correlated with hyperoxia and whith the greatest numbers of neutrophils. Additionally, reduced populations of neutrophils in the OVA + O_2_ group, compared with the OVA group, correlated with reduced Nrf2 levels. We speculate that the low expression of Nrf2 reveals a relationship between reductions to the neutrophil population by oxidative damage (with reduced total numbers of cells present) because oxidative damage with increased levels of malondialdehyde was observed only in the OVA + O_2_ group. These findings are consistent with previous studies conducted by our group that has shown the potential of the hyperoxia to reduce the population of inflammatory cells 102, as well as inducing apoptosis mainly through oxidative stress 103.

There is a hypothesis suggesting that hyperoxia could induce changes in naive TH cell phenotypes into TH17 cells through expression of IL-17 in pulmonary tissues. This hypothesis is based on the effects of ROS as important mediators of cell differentiation and immune modulation 104. We reasoned that hyperoxia could significantly alter the microenvironment of the inflammatory site and, consequently, induce increased IL-17 expression in lung tissue. This is consistent with the known effects of hyperoxia, including the induction of expression of IL-6 105–107, Nrf2, MPO, IFN- γ, IL-1α, IL-4, IL-5, IL-6, IL-13, IL-17, TNF-α, VEGF, MIP-2α, MIG, CCL2, LIF, KC (IP)-10 108, and TGF-β 109–114. In particular, IL-6 and TGF-β are precursor molecules (in mice) to polarization of naive TH cells into TH17.

The TH1/TH2 paradigm is not new 115. However, the new TH1/TH2/TH17 paradigm 116, and specifically the role of TH17 cells in adaptive immune response in asthma, is still being elucidated 117–121. Only elderly mice develop neutrophil influx into the airways concomitant with TH17 immune responses following exposure to an allergen (dust mites) 122. The amplification, maintenance and differentiation of naive TH to TH17 cells has been related to the activation of TGF-β, IL-6, and IL-21 123,124, and RORγt transcription factor 39,125. In the present study, hyperoxia induced the phenotypic polarization toward TH17 cells on OVA-induced inflammation, represented by increased IL-17 expression in pulmonary tissue in the OVA + O_2_ group. High levels of IL-17 expression accompanied increased numbers of neutrophils in the airways. We believe that the population of neutrophils has been attracted by the greater availability of IL-17A in airway.

Paradoxically, Dang et al. showed that high levels of IL-17 expression have also been observed in hypoxic conditions in the HIF1alpha dependent mannered. Together, these observations lead us to believe that the oxygen tension has a “bell-shape“ effect on Th17 cell differentiation, triggered by oxidative stress induced by both low as high O_2_ levels. Because our initial objective was to identify in situ expression of IL17, we specifically suggest that other future studies are needed to delineate precisely whether the differentiation Th17 cells under hyperoxic conditions is associated with intrinsic or extrinsic pathway signaling 126,127.

In histopathological analyses, we observed an intense infiltration into interstitial cells, especially in peribronchial regions, corroborating with previously findings 128,129. There were also evident areas of enlargement of peribronchial tissue associated with the OVA + O_2_ group. However, when hyperoxia was superimposed on ovalbumin-induced inflammation, a reduction of inflammatory cells in airways (bronchoalveolar lavage) was observed. A possible hypothesis is based on the presence of cells migrating into the airways with tissues dying by hyperoxia-induced oxidative damage, while cells of the pulmonary interstitial are preserved.

Together, our data suggest that hyperoxia promotes TH17 polarization in the immune response tissue damage associated with oxidative stress and the migration of inflammatory cells, particularly neutrophils, to the lung tissues and airways.
